# Supplementary material for: Estimating Mycobacterium tuberculosis transmission in a South African clinic: Spatiotemporal model based on person movements
Source: PLoS Comput Biol. 2025 Feb 18;21(2):e1012823. doi: 10.1371/journal.pcbi.1012823 (PMC11856658; doi:10.1371/journal.pcbi.1012823)
Supplement: S1 Text: Appendix — (PDF) [file pcbi.1012823.s001.pdf]

S1 Appendix

Estimating *Mycobacterium* tuberculosis transmission in a South African clinic: Spatiotemporal model based on person movements

Nicolas Banholzer[1], Keren Middelkoop[2], Juane Leukes[2], Ernest Weingartner[3], Remo Schmutz[1], Kathrin Zürcher[1], Matthias Egger[1,4,5], Robin Wood[2], and Lukas Fenner[1*]

[1]Institute of Social and Preventive Medicine, University of Bern, Bern, Switzerland

[2]Desmond Tutu HIV Centre, Department of Medicine, University of Cape Town, Cape Town, South Africa

[3]Institute for Sensors and Electronics, University of Applied Sciences and Arts Northwestern Switzerland, Windisch, Switzerland

[4]Population Health Sciences, University of Bristol, Bristol, United Kingdom

[5]Centre for Infectious Disease Epidemiology and Research, University of Cape Town, Cape Town, South Africa

[*]Corresponding author: lukas.fenner@ispm.unibe.ch

List of Texts

A Environmental and tracking data by study day **4**

B Processing of person-tracking data **6**

C Spatiotemporal modeling approach **9**

C.1 The Wells-Riley modeling framework 9

C.2 Model setup and notation 10

C.3 Quanta generation 11

C.4 Quanta diffusion 12

C.5 Quanta removal 13

C.6 Spatiotemporal quanta concentration 13

C.7 Illustrative example 13

D Simulations **16**

D.1 Setup and setting 16

D.2 Monte Carlo simulation 16

D.3 Modeling assumptions 17

E Additional simulation results **22**

F Discussion of factors influencing transmission risks **23**

F.1 Environmental factors 23

F.2 Pathogen-specific factors 23

F.3 Patient-specific factors 24

References **25**

List of Figures

A CO_2 levels by clinic area over time for each study day. Raw data at 1 min intervals averaged over a ten minute time window to reduce noise. 4

B Number of people by clinic area over time for each study day. Raw data at 1 second intervals averaged over a ten minute time window to reduce noise. 5
C Screenshot of the Shiny application used for reconnecting interrupted tracking IDs. . 8
D Example: Spatiotemporal quanta concentration in an indoor space 15
E Prior distribution for the quanta generation rate 19
F Prior distribution for the bacterial inactivation rate 20
G Counts of the daily number of diagnosed TB patients visiting the clinic 21
H Mean risk of infection per clinic attendee when varying assumptions about the number of infectious people in the clinic 22

A Environmental and tracking data by study day

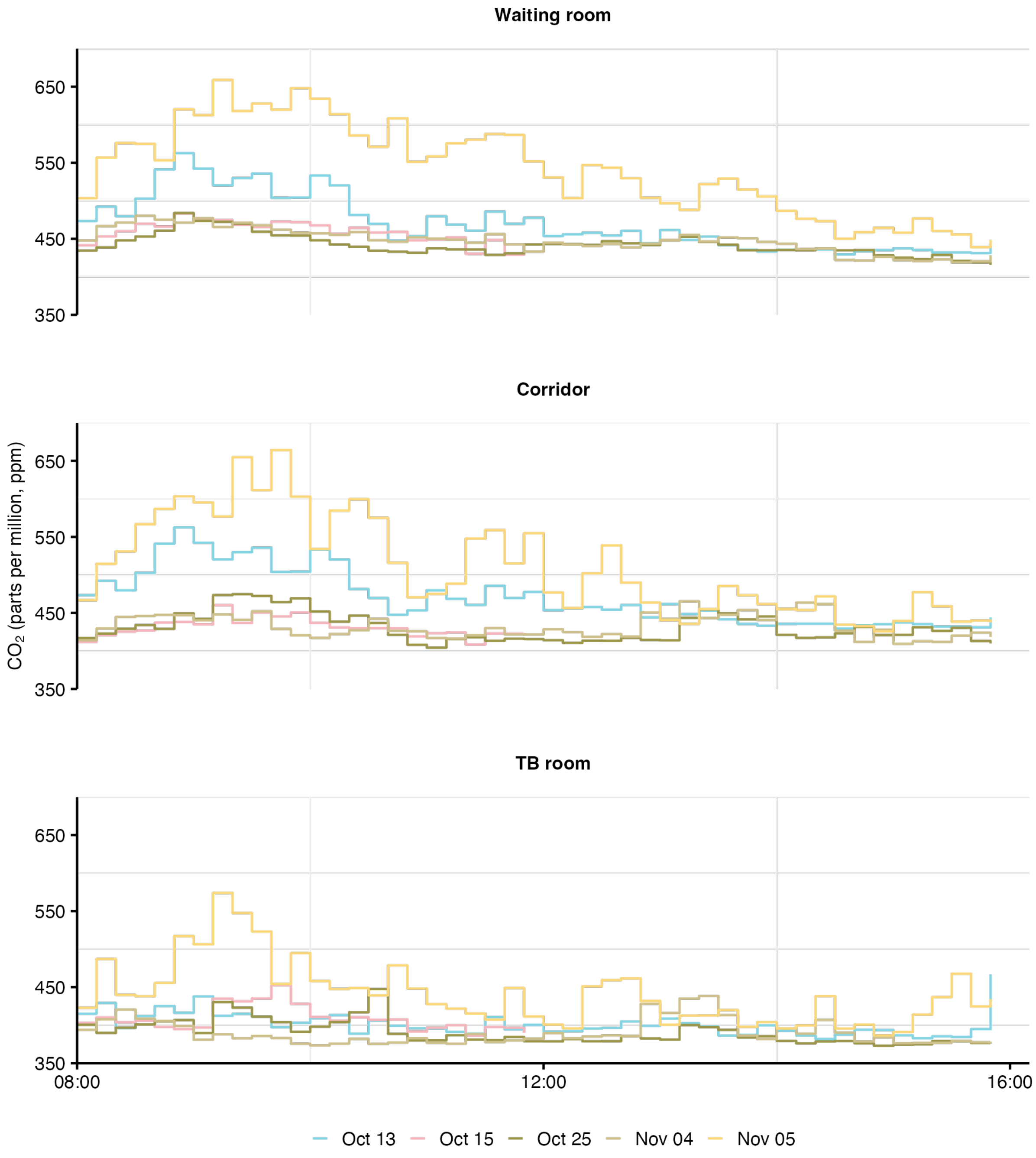

Figure A. CO_2 levels by clinic area over time for each study day. Raw data at 1 min intervals averaged over a ten minute time window to reduce noise.

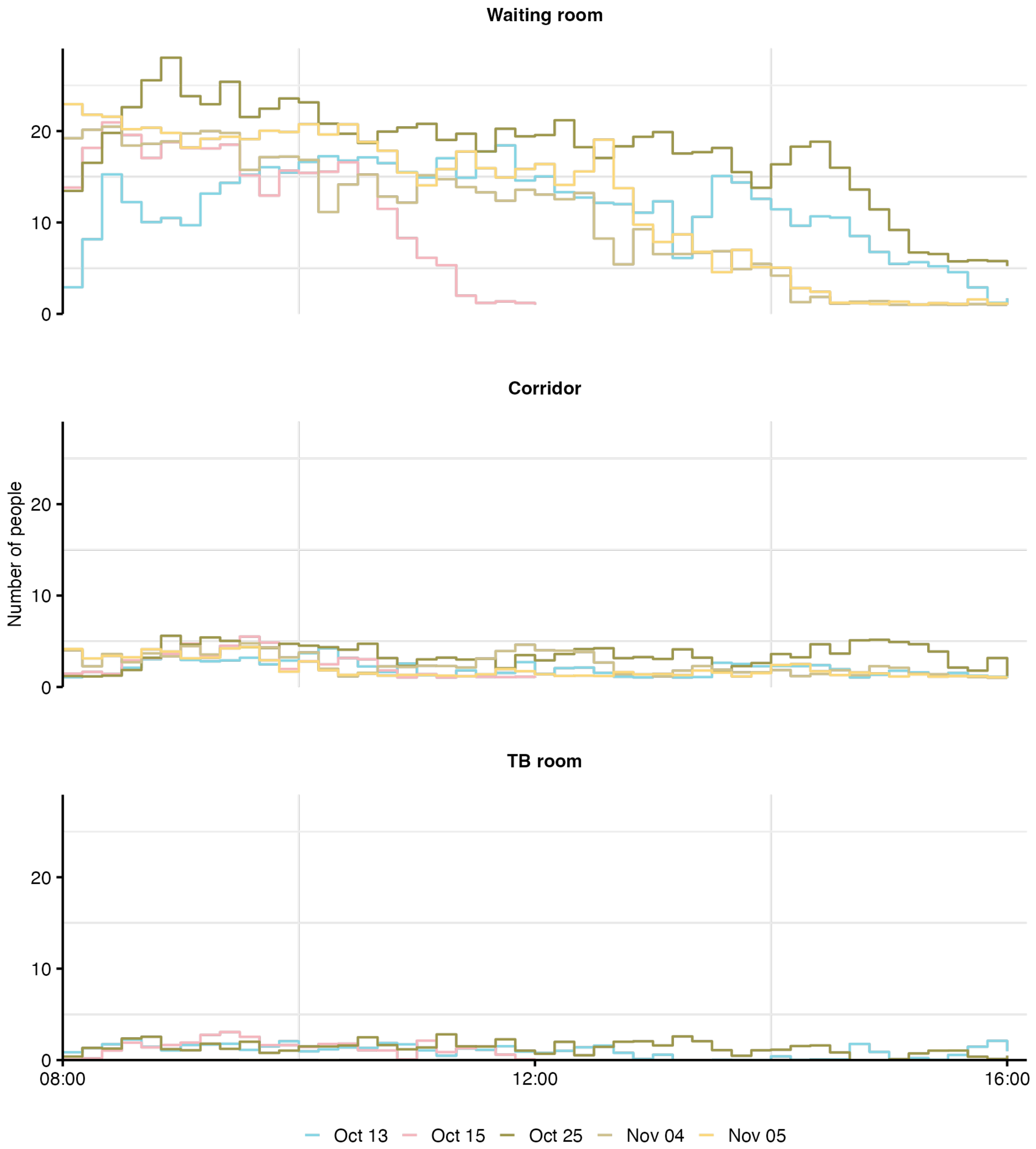

Figure B. Number of people by clinic area over time for each study day. Raw data at 1 second intervals averaged over a ten minute time window to reduce noise.

B Processing of person-tracking data

Person-tracking data required processing because many tracks were interrupted, for example, if the person i) moved outside the range of the video sensors and then re-entered, ii) bowed heavily downwards, or iii) was temporarily hidden behind another person or obstacle. In addition, moving objects such as opening doors may have been recognized as a person, introducing many extremely short tracks (total duration of less than 10 s and distance less than 2 m), which were removed.

We reconnected tracks that most likely belonged to the same person. First, we automatically matched tracks that were less than 10 s and 3 m apart while moving or less than 60 s and 0.5 m apart while sitting. Second, we manually reconnected the remaining tracks if they were likely from the same person. For that purpose, we developed a Shiny application and a matching manual (https://github.com/nbanho/clinic-transmission/wiki/Matching-Manual) to assist the students performing the matching.

The Shiny application is shown in Figure C. In the center panel, a floor plan of the clinic is shown in (x,y)-space. Different areas of the clinic are delineated by colors, e. g. seating areas are marked in purple and exit areas are marked in blue. Tracks are shown as lines with a open and crossed circles indicating the tracks' start and end, respectively. The currently selected track is shown in black, possible links in colors, and alternatives for the selected possible link in light gray.

Below the floor plan, a table is showing descriptive information about the possible links in relation to the currently selected track: (1) the patient ID of the selected track (Pid) and the observation ID of possible links (Oid), (2) the height difference at the end point of Pid and the start point of Oid, (3) the standing height (i. e. maximum height during the entire track) difference, (4) the duration of Oid, (5) the time difference, and (6) the distance between the end point of Pid and the start point of Oid. Possible links are sorted in ascending order by the time difference.

The Shiny application's left panel shows descriptive information about the loaded file, the date of the tracking data, the initial number of IDs before any manual linkages, the current number of IDs after considering manual linkages, and the current number of made linkages. It further shows the current Pid with shortcuts to move to the next or previous Pid, or the next or previous Pid at the entrance. Linkages can be done either forward or backward in time. The total duration of the Pid track (time in the clinic) is shown together with the number of connected tracks within Pid.

The sliding bars allow filtering of possible links (Oid) according to difference in time, distance, or height, with quick filters to switch between common thresholds. The first dropdown menu selects the Oid for which alternative matches should be shown in the floor plan. The idea is to prevent false linkages when there are more plausible alternatives for the Oid. A connection is made by selecting the Oid in the "Link with IDs" dropdown menu and then clicking "Link IDs". After that, the app updates the floor plan, matching table, and descriptive information. Erroneous links can be revoked by clicking "Unlink last ID", if the last link in time should be revoked, or by clicking "Unlink first ID", if the first link in time should be revoked. If the Pid could not be connected further, its track can be finished by entering a label and clicking "Label/End track". The person-tracking data is then updated with the reconnected track and saved automatically to the file shown at the bottom.

We created the following labels to denote finished tracks:

- **Clean**: A clean track throughout the clinic with known entrance and exit.
- **Staff**: A track moving in- and out of the staff room.
- **Noise**: A short track (≤1 min) without entrance or exit.
- **Lost enter**: A long track (>1 min) without entrance.
- **Lost exit**: A long track (>1 min) without exit.
- **Lost both**: A long track (>1 min) without entrance and without exit.

We obtained 3,319 tracks after preprocessing. Further exclusion of relatively short tracks <5 min resulted in 1,563 tracks used for analysis: 209 (13%) labeled as "Clean", 125 (8%) as "Staff", 213 (14%) as "Lost enter", 246 (16%) as "Lost exit", and 770 (49%) as "Lost both".

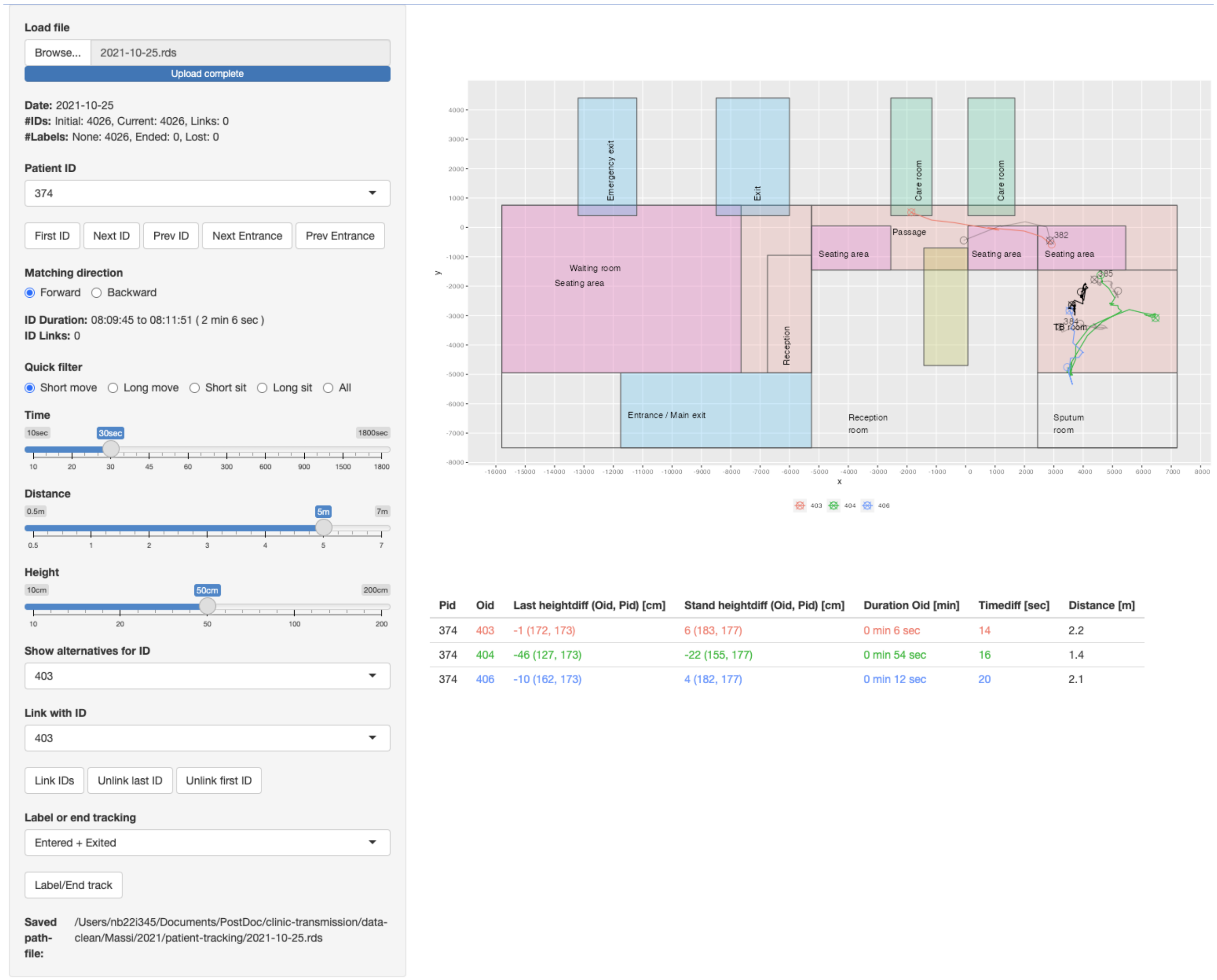

Figure C. Screenshot of the Shiny application used for reconnecting interrupted tracking IDs.

C Spatiotemporal modeling approach

C.1 The Wells-Riley modeling framework

We build upon the Wells-Riley model[1], which is an established epidemiological model to estimate the risk of infection. The model estimates the risk of airborne indoor transmission as

$$P = \frac{C}{S} = 1 - \exp\left(-\frac{Ipqt}{Q}\right), \tag{1}$$

where P is the probability of infection, C is the number of diseased cases, S is the number of susceptible cases, I is the number of infectious people in the indoor space, p is the breathing rate ($\mathrm{m^3\,h^{-1}}$), q is the quantum (infectious dose) generation rate ($\mathrm{quanta\,h^{-1}}$), t is the exposure time (s), and Q is the ventilation rate ($\mathrm{m^3\,h^{-1}}$).

The unknown parameter q is not the actual number of infectious particles in the air. It rather represents the dose of infectious particles that corresponds to a certain probability of infection with a Poisson relationship, e. g. one quantum corresponds to $P = 1 - \exp(1) = 63\%$ risk of infection[2]. It is an attempt to consider the stochastic behavior of airborne transmission, since the number of infectious particles that cause an infection is unknown. Moreover, the risk of transmission depends on the environment, characteristics of the pathogen, and characteristics of the individual. For example, the type of respiratory activity (e. g. breathing, coughing, or sneezing) determines the site in the respiratory tract where the exhaled particles are generated, which in turn influences the particle size distribution[3]. Smaller particles reach deeper into the lungs of the susceptible person[4], which increases the probability of infection.

While considering the stochastic behavior of airborne transmission, the Wells-Riley model makes two simplifying assumptions. First, it assumes a well-mixed airspace, which means that the generated quanta disperse immediately and uniformly in the indoor airspace. In other words, the exposure to infectious quanta is the same regardless of the location of the infectious and susceptible individual. Second, the Wells-Riley model assumes steady-state conditions, which means that the quanta concentration and the outdoor air supply rate are constant over time. In other words, the risk of infection for each susceptible only varies with the duration of exposure (the time spent in the indoor space).

Rudnick and Milton[2] modified the Wells-Riley model to relax the steady-state assumption. They use CO_2 as a biomarker for exhaled breath to compute the outdoor air supply rate, which is otherwise difficult to measure. Their modified equation is

$$P = \frac{C}{S} = 1 - \exp\left(-\frac{\bar{f}Iqt}{n}\right), \tag{2}$$

where $f = \frac{CO^{\mathrm{I}} - CO^{\mathrm{O}}}{CO^{\mathrm{A}}}$ is the fraction of indoor air that is exhaled breath, which is computed based on the CO_2 level in indoor air CO^{I}, outdoor air CO^{O}, and exhaled breath CO^{A} (in parts per million [ppm], respectively).

The extension by Rudnick and Milton still assumes a well-mixed airspace. We relax this assumption by modeling spatial variation in the concentration of infectious quanta. We then compute the risk of infection for susceptible individuals using the Wells-Riley equation considering their cumulative exposure to infectious quanta over space and time as

$$P = \sum_{c} \sum_{t} N_{c,t} \cdot \mathbb{I}_{c,t} \cdot p, \tag{3}$$

where N is the quanta concentration in a specific area of the indoor space c at time t (quanta m^{-3}), $\mathbb{I}$ indicates whether the individual was in c at t, and p is the breathing rate ($\mathrm{m}^3\,\mathrm{h}^{-1}$). Modeling of the spatiotemporal quanta concentration can subsequently be divided into three processes

C.3 **Quanta generation**: Infectious particles generated at the infectious individual's location.

C.4 **Quanta diffusion**: Pathogen-carrying particles disperse in the indoor space.

C.5 **Quanta removal**: Contaminated indoor air is replaced with fresh outdoor air.

We describe each process in greater detail in the following, but first we introduce some notation.

C.2 Model setup and notation

We assume that quanta concentration is well-mixed vertically and divide the airspace into a 2-dimensional grid with cells $c = 1, \ldots, C$, where (x_c, y_c) refer to the center of cell c. The grid provides a discrete approximation to the continuously varying quanta concentration in the indoor space, with the number of grid cells $C = n_x \cdot n_y$ determining the spatial resolution. We denote the following variables and modeling parameters:

- $N_{c,t}$: quanta concentration in $c = 1, \dots, C$ at $t = 1, \dots T$ (in seconds)
- $I_{c,t}$: number of infectious individuals in c at t
- q: quanta generation rate (quanta s^{-1})
- D: diffusion constant (m^2 s^{-1})
- AER: air change rate (s^{-1})
- V_c: volume of the cell (m^3)
- CO^{I}: CO_2 mixing ratio in indoor air (parts per million, ppm)
- CO^{O}: CO_2 mixing ratio in outdoor air (ppm)
- G: average CO_2 generation rate per person ($L \cdot min^{-1} \cdot person^{-1}$)
- n_t: number of individuals in the airspace at time t
- λ: bacterial inactivation rate (s^{-1})
- k: gravitational settling rate (s^{-1})
- p: breathing rate ($m^3\, s^{-1}$)

C.3 Quanta generation

The number of quanta generated by infectious individuals at time t in cell c is

$$I_{c,t} \cdot q \ . \tag{4}$$

For now, we assume that the initially generated quanta is confined to the infectious individual's cell, reflecting previous findings that the concentration of pathogen-laden aerosols is typically highest near the infectious source[4–7]. Although coughing and sneezing can expel the aerosols further away from the individual, these activities are much less frequent than breathing, which may thus contribute more infectious particles[8]. Note, however, that the model can also accommodate assumptions about a larger initial spread of quanta.

C.4 Quanta diffusion

Absent measurements of airflow, we assume that aerosols diffuse uniformly in the indoor air, approaching a well-mixed airspace over time (the steady state). We assume that the quanta diffuse radially in (x,y)-direction towards locations where the concentration is lower. The diffusion equation is given by

$$\frac{\delta N_{c,t}}{\delta t} = D\Delta N_{c,t}, \tag{5}$$

where Δ is the Laplace operator and D is the diffusion constant. We solve this equation with a standard second-order finite difference model using the Euler method with a 5-point stencil.

The diffusion constant D is unknown. We approximate it using an empirical relationship between the eddy diffusion coefficient $K \approx D$ ($\mathrm{m^2\,s^{-1}}$) and the outdoor air exchange rate AER for CO_2 particles[9,10]

$$K = (0.52 \cdot AER + 8.61 \cdot 10^{-5} \cdot \mathrm{s}^{-1}) \cdot V^{\frac{2}{3}}, \tag{6}$$

where V is the volume of the entire space (m^3). Implicitly, we assume that pathogen-carrying particles spread with a similar speed than CO_2 particles, which may overestimate the diffusion constant because CO_2 particles are smaller than *Mtb*-carrying particles.

The AER is not directly observed but can be estimated from indoor CO_2 levels and room occupancy. Under steady-state conditions (i. e. CO_2 levels reaching a steady-state mixing ratio), the AER can be computed as

$$AER = \frac{6 \cdot 10^4 \cdot n \cdot G}{V \cdot (CO^{\mathrm{I}} - CO^{\mathrm{O}})}, \tag{7}$$

where n is the number of people in the airspace, G is the average CO_2 generation rate per person, CO^{I} is the CO_2 mixing ratio in indoor and CO^{O} is the mixing ratio in outdoor air[11]. However, in a primary care clinic, room occupancy varies continuously and thus it may be difficult to determine the steady-state CO_2 mixing ratio. Therefore, we determine AER from time-varying CO_2 levels and

room occupancy using a transient mass balance model of the form[11]

$$CO^{\mathrm{I}}_{t+1} = \frac{6\cdot 10^4 \cdot n_t + G}{Q} \cdot (1-\exp(-Q/V\Delta t)) + (CO^{\mathrm{I}}_t - CO^{\mathrm{O}}) \cdot \exp(-Q/V\Delta t) + CO^{\mathrm{O}}, \tag{8}$$

where Q is the outdoor air supply rate ($\mathrm{m}^3\,\mathrm{s}^{-1} \cdot \frac{1}{3600}$) and $AER = Q/V$. We numerically solve the transient mass balance model with the limited memory Broyden–Fletcher–Goldfarb–Shanno algorithm (L-BFGS), a quasi-Newton method, as implemented in the statistical software R[12]. The optimal solution is the outdoor air supply rate Q that minimizes the root mean square error. We also follow recommendations to fit the outdoor CO_2 mixing ratio with reasonable constraints on $CO^{\mathrm{O}} = [300, 600]\,\mathrm{ppm}$[11].

C.5 Quanta removal

Infectious quanta may be removed from the air through outdoor air exchange, bacterial inactivation, or gravitational settling. The removal rate is thus the sum of the outdoor air exchange rate AER, the bacterial inactivation rate λ for *Mtb*, and the gravitational settling rate k for *Mtb*-carrying particles, i. e.

$$AER + \lambda + k \ . \tag{9}$$

C.6 Spatiotemporal quanta concentration

Combining Equations 4, 5, and 9, the quanta concentration can be computed as

$$\underbrace{N_{c,t}}_{\text{new conc.}} = \underbrace{\left(D\Delta(\underbrace{N_{c,t-1}}_{\text{old conc.}} + \underbrace{I_{c,t} \cdot q}_{\text{generation}} \cdot V_c^{-1}) \right)}_{\text{diffusion}} \cdot \underbrace{\exp\left(-(AER_t + \lambda + k\right)}_{\text{removal}}, \tag{10}$$

where V_c is the volume of cell c.

C.7 Illustrative example

We illustrate our modeling approach with an example. Consider a room with a volume V=150 m^3 (length = 10 m, breadth = 5 m, height = 3 m), which we rasterize into a grid of $C = 40 \cdot 20 = 800$ cells with an area of $(0.25\,\mathrm{m})^2$. We assume that quanta generation is confined to the cell where the

infectious individual is located as well as the first neighboring cells. We monitor the room from 8 am to 12 am and consider one infectious individual generating quanta continuously for one hour between 8 am and 9 am in the middle of the room in cell (20,10), which means that quanta is generated in cells ($x_c = 20 \pm 1, y_c = 10 \pm 1$). We assume a quanta generation rate of $q = 2$ quanta h^{-1} and a bacterial inactivation rate of $\lambda = 0\,\mathrm{h}^{-1}$. We further assume a constant outdoor air exchange rate of $AER = 1\,\mathrm{h}^{-1}$, corresponding to a diffusion constant of $0.015\,\mathrm{m}^2\,\mathrm{s}^{-1}$.

Figure D shows the spatiotemporal quanta concentration. The quanta concentration increases over time and is higher near the infectious individual in the room's center (Figure Da, top panel). After the individual has left the room, the quanta diffuses within minutes and most quanta is removed by 10 am through outdoor air exchange (Figure Da, bottom panel). Spatial variation in the quanta concentration peaks at 9 am, right before the individual leaves the space, and 15 min after that a well-mixed airspace has been reached through quanta diffusion (Figure Db).

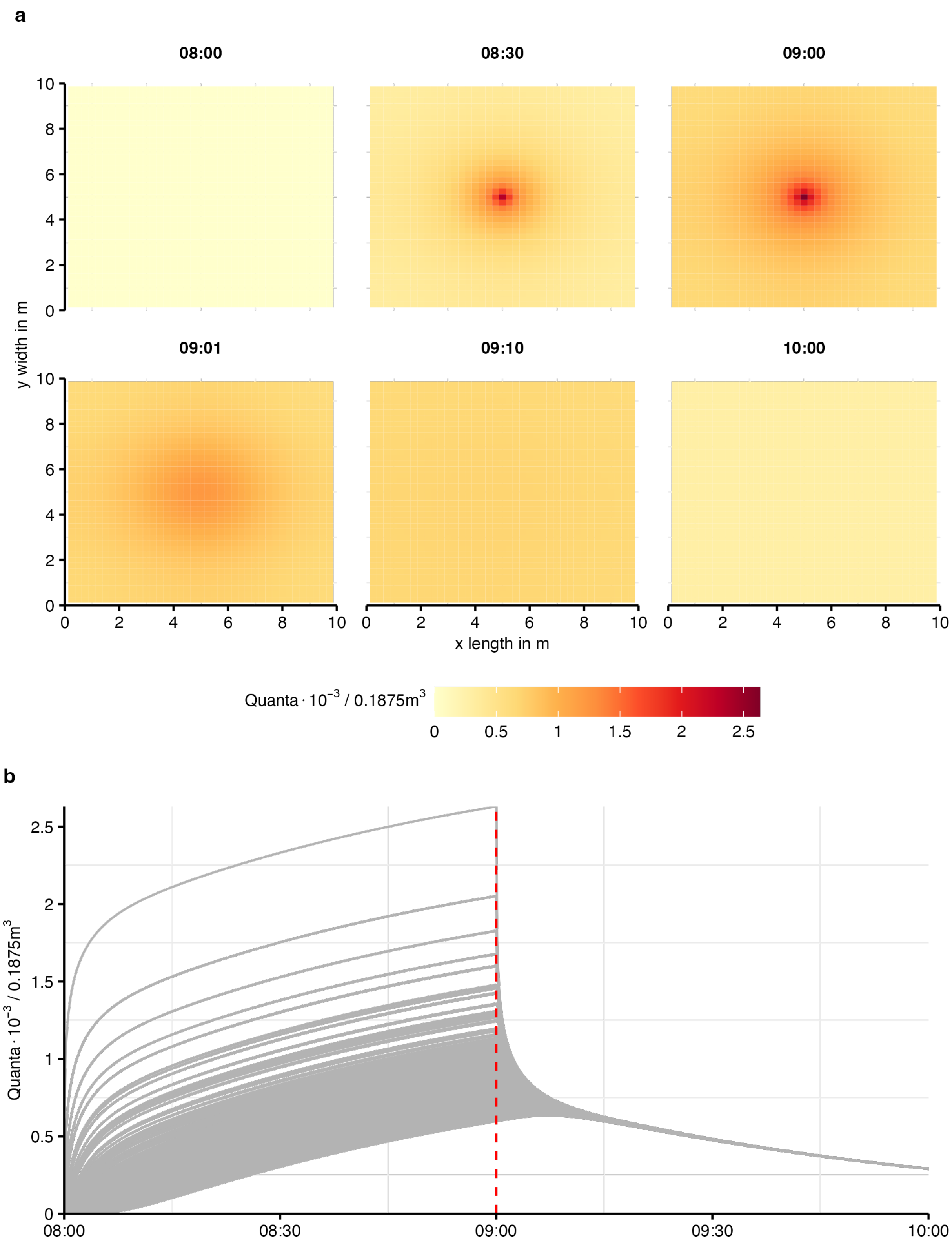

Figure D. Example: Spatiotemporal quanta concentration in an indoor space between 8 am and 10 am following the generation of quanta by an infectious individual between 8 am and 9 am in the center of the room. **(a)** Spatial quanta concentration at selected time points and **(b)** quanta concentration over time for the 800 cells into which the indoor space was rasterized.

D Simulations

D.1 Setup and setting

We divide the clinic into three spaces: the waiting room (length = 10.55 m × breadth = 5.50 m × height = 3.00 m), the corridor (length = 12.45 m × breadth = 2.20 m × height = 2.50 m), and the TB room (length = 4.75 m × breadth = 3.50 m × height = 3.00 m). Each room is discretized into a grid of cubic cells, with each cell covering a squared area of $(0.25\,\mathrm{m})^2$, corresponding to a diagonal cell length of $\sqrt{0.25^2 + 0.25^2} = 0.35\,\mathrm{m}$. The volume of the cell depends on the room height.

Although the door between the waiting room and corridor was usually not closed, we consider these rooms to be separate to facilitate modeling. We acknowledge the possibility for aerosolized *Mtb* to spread from the waiting room to the corridor, and vice versa, which is not considered.

We model the spatiotemporal quanta concentration during clinic hours from 8 am to 4 pm and compare the modeled concentration by daytime, i. e. morning (8 am to 12 am) and afternoon (12 am to 4 pm). Correspondingly, we estimate the outdoor air exchange rate by daytime, considering potential differences in ventilation conditions between the morning and afternoon. We update the quanta concentration every second from $t = 1, \ldots, T$, corresponding to the frequency of the person-tracking data. We set the quanta concentration at $t = 0$ to zero, i. e. $N_{r,c,0} = 0 \ \ \forall r, c$. That is, we assume that all quanta from the previous day has been removed from the air before the start of the following clinic day.

D.2 Monte Carlo simulation

We use Monte Carlo simulation to model the quanta concentration and estimate the individual risk of infection, considering uncertainty in several modeling parameters. Each simulation consists of the following steps:

1. **Sample uncertain modeling parameters**: Undiagnosed TB patients among clinic attendees, quanta generation rate, mask reduction rate, bacterial inactivation rate, and gravitational settling rate.

2. **Model quanta concentration**: Based on the generation of infectious quanta by diagnosed and undiagnosed TB patients, we compute the quanta concentration in each room and cell

over time.

3. **Estimate risk of infection**: Based on their cumulative quanta exposure, we estimate the risk of infection for each clinic attendee using the Wells-Riley equation.

Many clinic attendees can be considered potential undiagnosed TB patients and prior uncertainty in the quanta generation, mask reduction, bacterial inactivation, and gravitational settling rate is large. To reflect this uncertainty, we perform 5,000 Monte Carlo simulations and compute the individual risk of infection as the mean across simulations.

D.3 Modeling assumptions

Initial spread

The initial spread, referred to as the propagation distance, is the maximum distance from the source to the end of the exhaled air before dispersion. An analysis of exhalation activities suggest a propagation distance of 0.7 m[13], but larger distances may be reached depending on the activity (i. e. breathing, speaking, coughing). Face masks reduce the propagation distance both by blocking (the mask capturing particles) and deflecting (leakage of particles on the sides of the mask) particles[14–16]. Therefore, we assume that mask wearing confines the quanta generation only to the cell where the infectious individual is located, hence our choice of the cell length corresponding to a propagation distance of 0.35 m. Without masks, we assume the unmitigated propagation distance of 0.7 m (the diagonal lengths of two cells). Since the direction of propagation is not observed, we assume uniform spread to all neighboring cells, i. e. the generated quanta is distributed evenly among the neighboring cells from where the infectious individual is present.

Quanta generation rate

Previous studies estimated the quanta generation rate of *Mtb* using the Wells-Riley model[17–21]. Andrews et al.[17] reported an estimate of 0.89 (quanta h^{-1}) (range from 0.44 to 5.69), close to the early estimate provided by Riley et al.[18] with 1.25 quanta h^{-1}. On the other end, Escombe et al.[19] arrived at an average estimate of 8.2 quanta h^{-1}, which, however, could be as high as 226 quanta h^{-1}. Lower rates were observed in non-multidrug-resistant (MDR), smear-negative patients and considerably higher ones in MDR, smear-positive TB patients[19]. Dharmadhikari et al.[21] also estimated a high average rate of 138 quanta h^{-1} in MDR TB patients, and Nardell et al.[20]

reported an estimate of 13 quanta h^{-1} for a single index case. The wide range of estimates within and between studies suggest that the emitted quanta varies considerably with the infectiousness of *Mtb* patients[22]. To consider such variation, Mikszewski et al.[23] used the bacterial load distribution in sputum to estimate the quanta generation rate with the approach developed by Buonanno et al.[24]. We have recently adopted this approach to derive quanta generation rate distributions for different activity levels in a school setting[25]. Here, we follow the same approach to derive a quanta generation rate distribution for sitting and walking activities, assuming 80% breathing and 20% speaking during both activities. The resulting prior distributions are shown in Figure E along with the estimates from the literature. The quanta generation rate follows a lognormal prior distribution with median 1.08 quanta h^{-1} (95%-credible interval [CrI] 0.003 quanta h^{-1} − 386 quanta h^{-1}) while sitting and 2.81 quanta h^{-1} (95%-CrI 0.008 quanta h^{-1} − 987 quanta h^{-1}) while walking. The activity is determined based on whether the person moves by more or less than 0.25 m (spatial distance between two recorded tracks of the same person).

Mask reduction rate

Face masks reduce the number of viral-laden aerosols that are exhaled into the air[26,27] and should thus lower the generation of infectious quanta. McCreesh et al. assumed a reduction by mask-wearing of 75% (95%-confidence interval (56% to 85%) based on the findings of Dharmadhikari et al.[21], which we translate into a Beta(24.9, 8.3) prior distribution for the mask reduction rate. We assume that masks were always correctly worn by all clinic attendees and staff during the study.

Bacterial inactivation rate

The bacterial inactivation rate for *Mtb* has not been precisely estimated. Loudon (1969)[28] report a half-life for aerosolized TB bacilli of 6 h ($\lambda = 0.12\,h^{-1}$). By contrast, Lever (2000)[29] reported a half-life of just 5 min ($\lambda = 8.3\,h^{-1}$). The difference could be explained by differences in humidity levels during the study[29]: Loudon (1969) and Lever (2000) studied survival in an environment with relative humidity of about 50% and 70%, respectively. Relative humidity in our study is closer to 50%, thus the estimate by Loudon (1969) may be more suitable for our study. Furthermore, Gannon (2007)[30] report a half-life of 1.5 h ($\lambda = 0.46\,h^{-1}$) at relative humidity of over 75%, yet the estimate is for *Mtb bovis*, and Klein (2014)[31] observe a 80% reduction within 30 min ($\lambda = 3.2\,h^{-1}$), which is closer to the estimate by Lever (2000). Considering the variation in these estimates, we model λ with

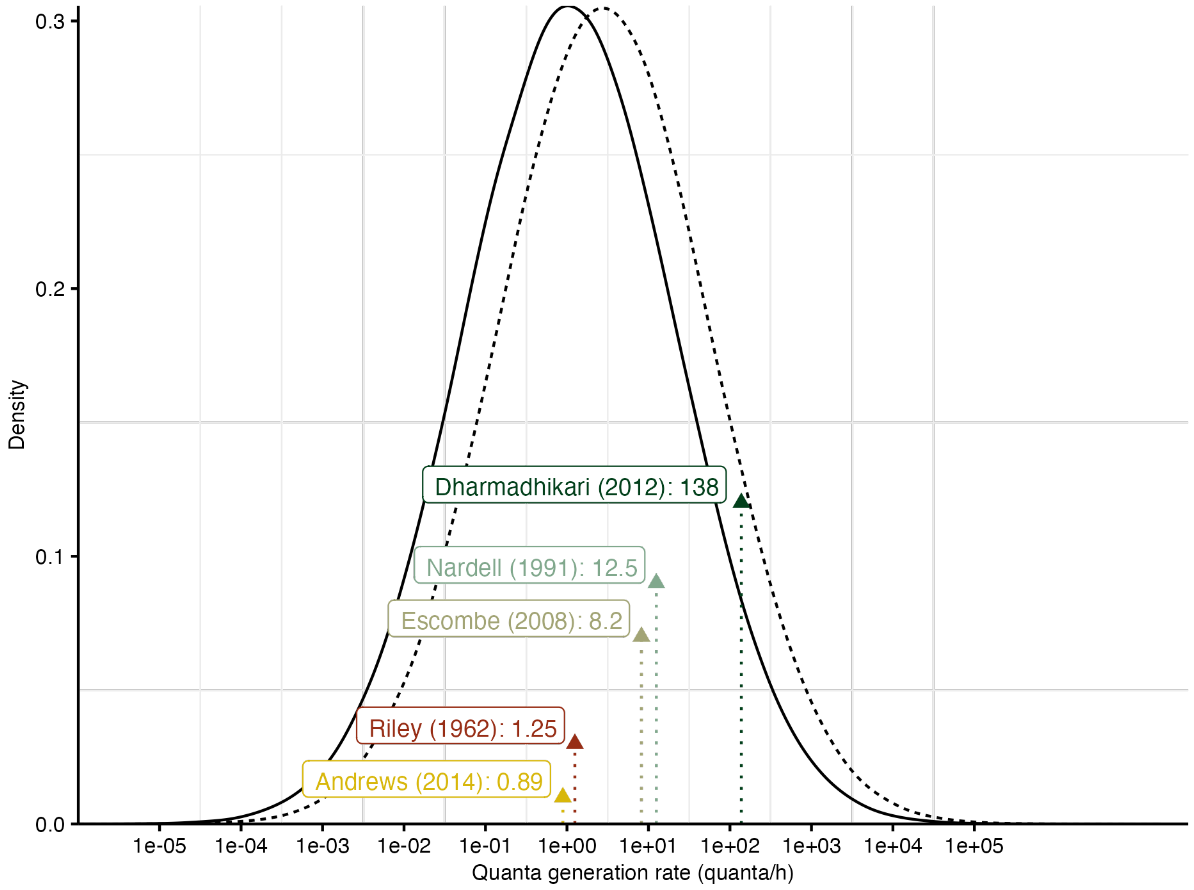

Figure E. Prior distribution (log scale) for the quanta generation rate (quanta h^{-1}) by activity level (sitting and walking), along with reported average estimates from the literature[17–21].

a Lognormal($\mu = \log 1, \sigma = 1$) distribution, with a median rate of $1\,h^{-1}$ (95%-CrI $0.1\,h^{-1} - 7.1\,h^{-1}$). The distribution is shown in Figure F along with the estimates from the literature.

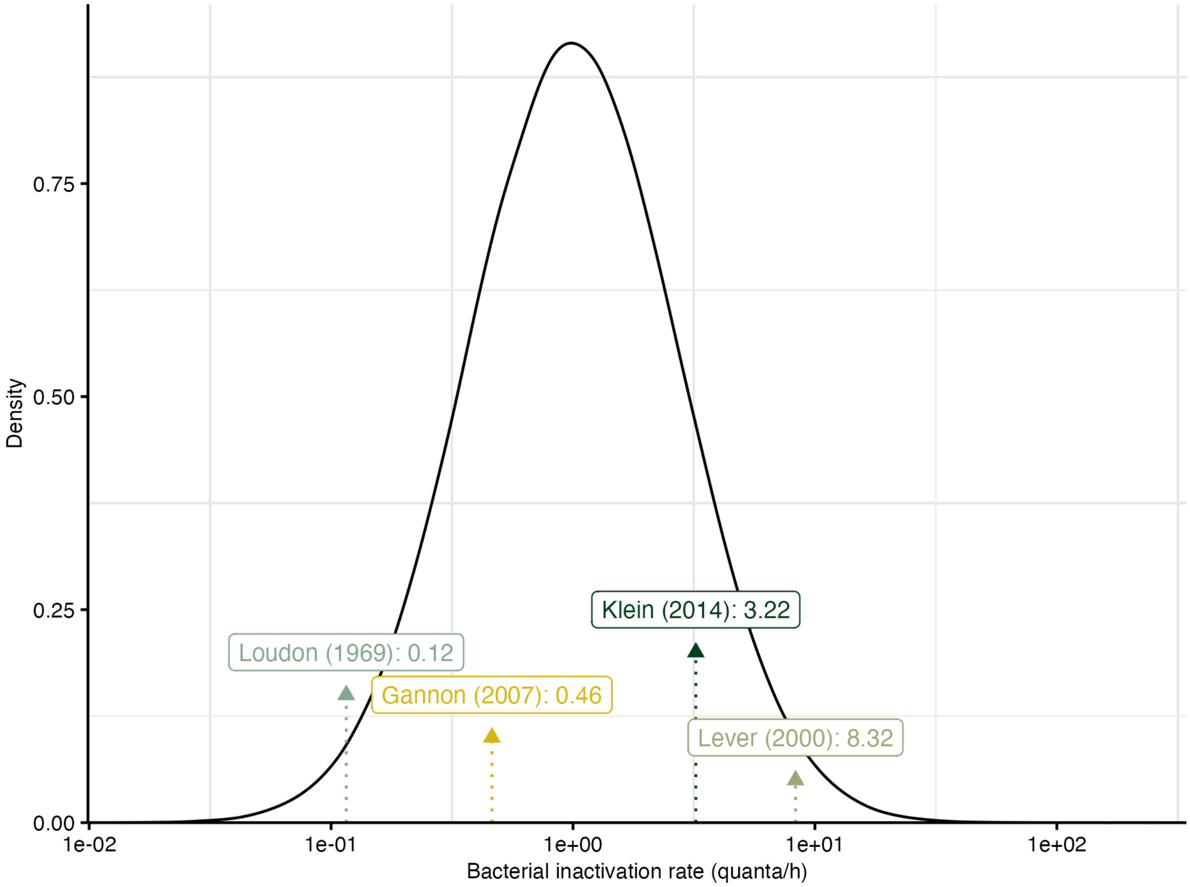

Figure F. Prior distribution (log scale) for the bacterial inactivation rate (quanta h^{-1}) of *Mtb* along with reported estimates from the literature[28–31].

Gravitational settling rate

Particles with *Mtb* in the size range of $1 - 7\mu m$[32] have a settling velocity of $3.5 \cdot 10^{-5} - 1.5 \cdot 10^{-3} m\,s^{-1}$ in still air[6]. Accordingly, we model the settling velocity using a Gamma distribution with mean $7.7 \cdot 10^{-4} m\,s^{-1}$ and standard deviation $3.7 \cdot 10^{-4} m\,s^{-1}$, which is slightly skewed towards slower settling velocities, reflecting a higher proportion of *Mtb* at the lower end of the size range[32]. Assuming a drop height of 1.7 m, the distribution for the gravitational settling rate has a median of 1.5 h^{-1} (95%-CrI 0.5 h^{-1} – 3.5 h^{-1}).

Number of undiagnosed TB patients

Undiagnosed (subclinical) TB patients may go undetected because they do not show symptoms according to the WHO 4-question symptom screen (cough, fever, weight loss, and night sweats)[33], or the diagnosis could not be made at first presentation based on existing tests[34]. Previous studies suggest that the number of undiagnosed (subclinical) TB patients is roughly similar to the number of diagnosed TB patients[33,35]. Therefore, we model the number of undiagnosed TB patients with a Multinomial distribution based on the counts of the daily number of diagnosed TB patients from October to November 2021 (Figure G). We consider all clinic attendees as potential undiagnosed TB patients and sample among them with equal probability. We further assume that undiagnosed and diagnosed TB patients have the same quanta generation rate.

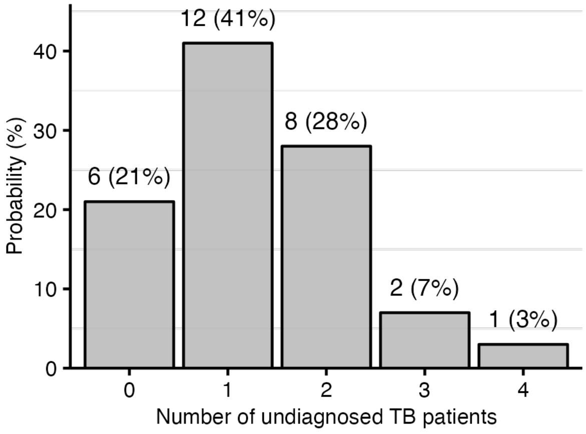

Figure G. Counts (%) of the daily number of diagnosed TB patients visiting the clinic. The number of diagnosed patients is modeled with a Multinomial distribution based on these counts.

Breathing rate

Adams (1993)[36] reports breathing rates for different activity levels. We use the average breathing rate for the following activity levels: sitting with $p = 0.51\,\mathrm{m^3\,h^{-1}}$ and walking (slowly at 2.5 mph) with $p = 1.33\,\mathrm{m^3\,h^{-1}}$.

E Additional simulation results

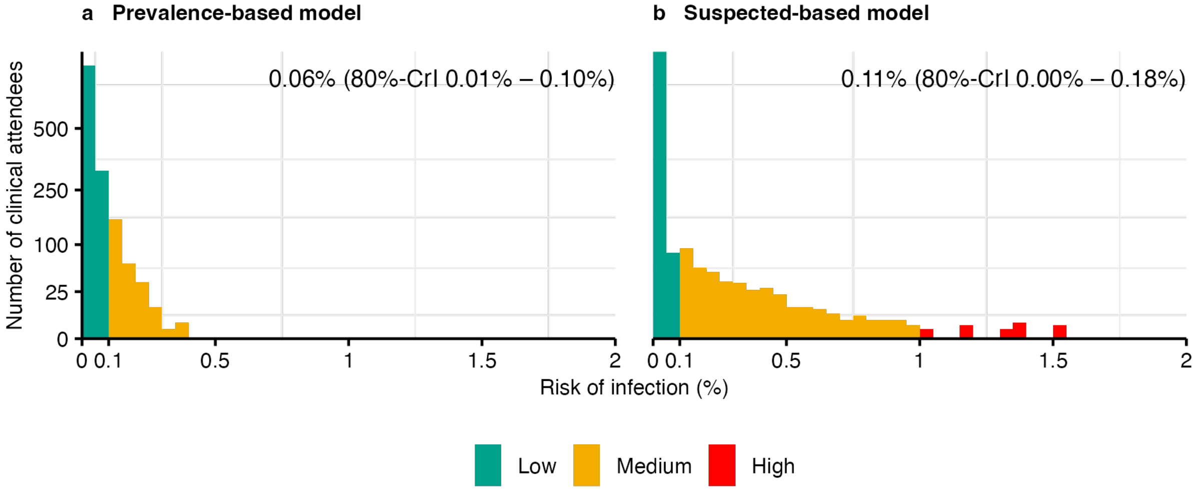

Figure H. Mean risk of infection per clinic attendee if **(a)** the number of infectious people in the clinic corresponded to the prevalence of TB in the South African population, or **(b)** the number of infectious people in the clinic corresponded to the number of bacteriologically confirmed TB patients and those suspected of having TB due to respiratory symptoms when attending the clinic. Low: <0.1%, Medium: 0.1-1%, High: >1% risk of infection.

F Discussion of factors influencing transmission risks

The generation, dispersion, and survival of infectious particles in the air is subject to considerable uncertainty. Therefore, Wells[37] introduced the concept of a "quantum" (infectious dose) to capture the stochastic nature of airborne transmission. A higher concentration of infectious quanta corresponds to a higher probability for an individual to get infected. The probability of infection is modeled with a Poisson relation and the Wells-Riley model considers multiple determinants for the generation and survival of infectious quanta. Nevertheless, transmission events are difficult to predict as the occurrence depends on multiple other factors that can broadly be categorized into (1) environmental factors, (2) physicochemical properties of the pathogen, and the (3) infectiousness of the source and susceptibility of the host.

F.1 Environmental factors

Environmental factors are somewhat considered by our model using CO_2 as a tracer gas to estimate the air exchange rate, based on which we model quanta diffusion and removal. The combination of clinical and person-tracking data further allows the spatial modeling of quanta generation. This makes our approach unique compared to existing modeling based on the Wells-Riley equation assuming a well-mixed airspace[1,2]. In our spatiotemporal model, we assume a radial diffusion of quanta, which may not reflect the actual airflow at the primary care clinic. The spatial dispersion of infectious particles is often simulated with computational fluid dynamics (CFD) models[6,38,39]. CFD models can simulate a variety of diffusion patterns, which help researchers understanding the influence of airflow. For example, measuring airflow can identify poorly ventilated indoor spaces where infectious particles circulate inside the room rather than getting replaced with fresh outdoor air[39]. However, airflow is rarely considered in modeling because it is difficult to assess in a naturally ventilated space, such as our primary care clinic, where the airflow depends on the outdoor wind direction and which doors and windows are open.

F.2 Pathogen-specific factors

Survival and dispersion of aerosols are further determined by the physicochemical properties of virus-laden aerosols such as particle size, viral load, and other chemical components[4]. Currently,

these stochastic properties are only indirectly incorporated in the Wells-Riley modeling framework via the quanta generation rate parameter[1,2]. It is challenging to consider these properties specifically, especially because they can be modified by environmental factors with often unclear, pathogen-specific effects[40–42,42–44]. For example, survival of bacteria in aerosols decreases with temperatures >24°C, but the effects of humidity are unclear[44]. On the one hand, a comparison between two studies suggested that longer airborne survival of *Mtb* could be attributed to lower relative humidity[28,29]. On the other hand, airborne *Mtb* was more likely detected in healthcare facilitates with higher relative humidity[45,46]. Ambient carbon dioxide levels may further influence aerostability[47].

F.3 Patient-specific factors

Previous studies show considerable variation in the generation of infectious quanta by TB infected patients[17,19]. We model such variation by using a wide prior distribution for the quanta generation rate. Our model could be easily extended to incorporate prior information on the infectiousness of each individual TB patient. In general, only patients with active pulmonary TB are able to produce infectious droplets[48]. Infectiousness could be determined based on the most recent sputum test result. Sputum smear-positive patients are more likely to transmit *Mtb* than sputum smear-negative patients[49–51]. HIV status could further inform both infectiousness and susceptibility. On the one hand, HIV-positive patients are more likely to have a negative sputum smear microscopy result than HIV-negative patients, due to the reduced lung cavitation and the fewer *Mtb* bacilli in their sputum[50,52]. On the other hand, HIV-positive patients are less prone to control the *Mtb* infection than HIV-negative patients, due to their reduced immune status[53–55], and therefore TB disease progresses faster and is often more severe in HIV-positive TB patients compared to HIV-negative ones.

References

1. Riley RL, O'Grady F. Airborne infection: Transmission and control. Macmillan; 1961.

2. Rudnick SN, Milton DK. Risk of indoor airborne infection transmission estimated from carbon dioxide concentration. Indoor Air. 2003;13(3):237–245. doi:10.1034/j.1600-0668.2003.00189.x.

3. Wei J, Li Y. Airborne spread of infectious agents in the indoor environment. Am J Infect Control. 2016;44(9, Supplement):S102–S108. doi:10.1016/j.ajic.2016.06.003.

4. Wang CC, Prather KA, Sznitman J, Jimenez JL, Lakdawala SS, Tufekci Z, et al. Airborne transmission of respiratory viruses. Science. 2021;373(6558):eabd9149. doi:10.1126/science.abd9149.

5. Morawska L, Allen J, Bahnfleth W, Bluyssen PM, Boerstra A, Buonanno G, et al. A paradigm shift to combat indoor respiratory infection. Science. 2021;372(6543):689–691. doi:10.1126/science.abg2025.

6. Vuorinen V, Aarnio M, Alava M, Alopaeus V, Atanasova N, Auvinen M, et al. Modelling aerosol transport and virus exposure with numerical simulations in relation to SARS-CoV-2 transmission by inhalation indoors. Saf Sci. 2020;130:104866. doi:10.1016/j.ssci.2020.104866.

7. Chen W, Zhang N, Wei J, Yen HL, Li Y. Short-range airborne route dominates exposure of respiratory infection during close contact. Build Environ. 2020;176:106859. doi:10.1016/j.buildenv.2020.106859.

8. Dinkele R, Gessner S, McKerry A, Leonard B, Leukes J, Seldon R, et al. Aerosolization of *Mycobacterium tuberculosis* by tidal breathing. Am J Respir Crit Care Med. 2022;206(2):206–216. doi:10.1164/rccm.202110-2378OC.

9. Cheng KC, Acevedo-Bolton V, Jiang RT, Klepeis NE, Ott WR, Fringer OB, et al. Modeling exposure close to air pollution sources in naturally ventilated residences: Association of turbulent diffusion coefficient with air change rate. Environ Sci Technol. 2011;45(9):4016–4022. doi:10.1021/es103080p.

10. Foat T, Drodge J, Nally J, Parker S. A relationship for the diffusion coefficient in eddy diffusion based indoor dispersion modelling. Build Environ. 2020;169:106591. doi:10.1016/j.buildenv.2019.106591.

11. Batterman S. Review and extension of CO2-based methods to determine ventilation rates with application to school classrooms. Int J Environ Res Public Health. 2017;14(2):145. doi:10.3390/ijerph14020145.

12. Byrd RH, Lu P, Nocedal J, Zhu C. A limited memory algorithm for bound constrained optimization. SIAM J Sci Comput. 1995;16(5):1190–1208. doi:10.1137/0916069.

13. Tang JW, Nicolle AD, Klettner CA, Pantelic J, Wang L, Suhaimi AB, et al. Airflow dynamics of human jets: Sneezing and breathing - Potential sources of infectious aerosols. PLoS ONE. 2013;8(4):e59970. doi:10.1371/journal.pone.0059970.

14. Tang JW, Liebner TJ, Craven BA, Settles GS. A schlieren optical study of the human cough with and without wearing masks for aerosol infection control. J R Soc Interface. 2009;6(Suppl 6):S727–S736. doi:10.1098/rsif.2009.0295.focus.

15. Hui DS, Chow BK, Chu L, Ng SS, Lee N, Gin T, et al. Exhaled air dispersion during coughing with and without wearing a surgical or N95 mask. PLOS ONE. 2012;7(12):e50845. doi:10.1371/journal.pone.0050845.

16. Mansour MM, Smaldone GC. Respiratory source control versus receiver protection: Impact of facemask fit. J Aerosol Med Pulm. 2013;26(3):131–137. doi:10.1089/jamp.2012.0998.

17. Andrews JR, Morrow C, Walensky RP, Wood R. Integrating social contact and environmental data in evaluating Tuberculosis transmission in a South African township. J Infect Dis. 2014;210(4):597–603. doi:10.1093/infdis/jiu138.

18. Riley RL, Mills CC, O'grady F, Sultan LU, Wittstadt F, Shivpuri DN. Infectiousness of air from a tuberculosis ward. Ultraviolet irradiation of infected air: Comparative infectiousness of different patients. Am Rev Respir Dis. 1962;85:511–525. doi:10.1164/arrd.1962.85.4.511.

19. Escombe AR, Moore DAJ, Gilman RH, Pan W, Navincopa M, Ticona E, et al. The infectiousness of tuberculosis patients coinfected with HIV. PLOS Med. 2008;5(9):e188. doi:10.1371/journal.pmed.0050188.

20. Nardell EA, Keegan J, Cheney SA, Etkind SC. Airborne infection: Theoretical limits of protection achievable by building ventilation. Am Rev Respir Dis. 1991;144(2):302–306. doi:10.1164/ajrccm/144.2.302.

21. Dharmadhikari AS, Mphahlele M, Stoltz A, Venter K, Mathebula R, Masotla T, et al. Surgical face masks worn by patients with multidrug-resistant tuberculosis: Impact on infectivity of air on a hospital ward. Am J Respir Crit Care Med. 2012;185(10):1104–1109. doi:10.1164/rccm.201107-1190OC.

22. Wurie FB, Lawn SD, Booth H, Sonnenberg P, Hayward AC. Bioaerosol production by patients with tuberculosis during normal tidal breathing: implications for transmission risk. Thorax. 2016;71(6):549–554. doi:10.1136/thoraxjnl-2015-207295.

23. Mikszewski A, Stabile L, Buonanno G, Morawska L. The airborne contagiousness of respiratory viruses: A comparative analysis and implications for mitigation. Geosci Front. 2021;13(6):101285. doi:10.1016/j.gsf.2021.101285.

24. Buonanno G, Morawska L, Stabile L. Quantitative assessment of the risk of airborne transmission of SARS-CoV-2 infection: Prospective and retrospective applications. Environ Int. 2020;145:106112. doi:10.1016/j.envint.2020.106112.

25. Banholzer N, Schmutz R, Middelkoop K, Hella J, Egger M, Wood R, et al. Airborne transmission risks of tuberculosis and COVID-19 in schools in South Africa, Switzerland, and Tanzania: Modeling of environmental data. PLOS Glob Public Health. 2024;4(1):e0002800. doi:10.1371/journal.pgph.0002800.

26. Milton DK, Fabian MP, Cowling BJ, Grantham ML, McDevitt JJ. Influenza virus aerosols in human exhaled breath: Particle size, culturability, and effect of surgical masks. PLOS Pathog. 2013;9(3):e1003205. doi:10.1371/journal.ppat.1003205.

27. Leung NHL, Chu DKW, Shiu EYC, Chan KH, McDevitt JJ, Hau BJP, et al. Respiratory virus shedding in exhaled breath and efficacy of face masks. Nat Med. 2020;26(5):676–680. doi:10.1038/s41591-020-0843-2.

28. Loudon RG, Bumgarner LR, Lacy J, Coffman GK. Aerial transmission of mycobacteria. Am Rev Respir Dis. 1969;100(2):165–171. doi:10.1164/arrd.1969.100.2.165.

29. Lever Ms, Williams A, Bennett Am. Survival of mycobacterial species in aerosols generated from artificial saliva. Lett Appl Microbiol. 2000;31(3):238–241. doi:10.1046/j.1365-2672.2000.00807.x.

30. Gannon BW, Hayes CM, Roe JM. Survival rate of airborne Mycobacterium bovis. Res J Vet Sci. 2007;82(2):169–172. doi:10.1016/j.rvsc.2006.07.011.

31. Klein K, Yang Z. Comparison of ambient air survival of Mycobacterium tuberculosis clinical strains associated with different epidemiological phenotypes. Int J Mycobacteriol. 2014;3(3):211–213. doi:10.1016/j.ijmyco.2014.04.002.

32. Fennelly KP. Particle sizes of infectious aerosols: implications for infection control. Lancet Respir Med. 2020;8(9):914–924. doi:10.1016/S2213-2600(20)30323-4.

33. Berhanu RH, Lebina L, Nonyane BAS, Milovanovic M, Kinghorn A, Connell L, et al. Yield of facility-based targeted universal testing for Tuberculosis with Xpert and Mycobacterial culture in high-risk groups attending primary care facilities in South Africa. Clin Infect Dis. 2023;p. ciac965. doi:10.1093/cid/ciac965.

34. Patterson B, Dinkele R, Gessner S, Koch A, Hoosen Z, January V, et al. Aerosolization of viable *Mycobacterium tuberculosis* bacilli by tuberculosis clinic attendees independent of sputum-Xpert Ultra status. Proc Natl Acad Sci. 2024;121(12):e2314813121. doi:10.1073/pnas.2314813121.

35. Moyo S, Ismail F, Walt MVd, Ismail N, Mkhondo N, Dlamini S, et al. Prevalence of bacteriologically confirmed pulmonary tuberculosis in South Africa, 2017–19: A multistage, cluster-based, cross-sectional survey. Lancet Infect Dis. 2022;22(8):1172–1180. doi:10.1016/S1473-3099(22)00149-9.

36. Adams WC. Measurement of breathing rate and volume in routinely performed daily activities [final report]. Human Performance Laboratory, Physical Education Department, University of California, Davis; 1993. A033-205. Available from: https://ww2.arb.ca.gov/sites/default/files/classic/research/apr/past/a033-205.pdf.

37. Wells WF. Airborne contagion and air hygiene: An ecological study of droplet infections. Commonwealth Fund; 1955.

38. Jung J, Lee J, Jo S, Bae S, Kim JY, Cha HH, et al. Nosocomial outbreak of COVID-19 in a hematologic ward. Infect Chemother. 2021;53(2):332. doi:10.3947/ic.2021.0046.

39. Li Y, Qian H, Hang J, Chen X, Cheng P, Ling H, et al. Probable airborne transmission of SARS-CoV-2 in a poorly ventilated restaurant. Build Environ. 2021;196:107788.

doi:10.1016/j.buildenv.2021.107788.

40. Songer JR. Influence of relative humidity on the survival of some airborne viruses. J Appl Micriobol. 1967;15(1):35–42. doi:10.1128/am.15.1.35-42.1967.

41. Chan KH, Peiris JSM, Lam SY, Poon LLM, Yuen KY, Seto WH. The effects of temperature and relative humidity on the viability of the SARS Coronavirus. Adv Virol. 2011;2011:e734690. doi:10.1155/2011/734690.

42. Fernstrom A, Goldblatt M. Aerobiology and its role in the transmission of infectious diseases. J Pathog. 2013;2013:e493960. doi:10.1155/2013/493960.

43. Cox CS, Wathes CM. The aerobiological pathway of microorganisms. In: Bioaerosols Handbook. CRC Press; 1995. p. 77–99.

44. Tang JW. The effect of environmental parameters on the survival of airborne infectious agents. J R Soc Interface. 2009;6(Suppl 6):S737–S746. doi:10.1098/rsif.2009.0227.focus.

45. Sornboot J, Aekplakorn W, Ramasoota P, Bualert S, Tumwasorn S, Jiamjarasrangsi W. Detection of airborne Mycobacterium tuberculosis complex in high-risk areas of health care facilities in Thailand. Int J Tuberc Lung Dis. 2019;23(4):465–473. doi:10.5588/ijtld.18.0218.

46. Matuka DO, Duba T, Ngcobo Z, Made F, Muleba L, Nthoke T, et al. Occupational risk of airborne Mycobacterium tuberculosis exposure: A situational analysis in a three-tier public healthcare system in South Africa. Int J Environ Res Public Health. 2021;18(19):10130. doi:10.3390/ijerph181910130.

47. Haddrell A, Oswin H, Otero-Fernandez M, Robinson JF, Cogan T, Alexander R, et al. Ambient carbon dioxide concentration correlates with SARS-CoV-2 aerostability and infection risk. Nat Commun. 2024;15:3487. doi:10.1038/s41467-024-47777-5.

48. Rieder HL. Disease IUATaL, editor. Epidemiologic basis of tuberculosis control. International Union Against Tuberculosis and Lung Disease; 1999.

49. Shaw JB, Wynn-Williams N. Infectivity of pulmonary tuberculosis in relation to sputum status. Am Rev Tuberc. 1954;69(5):724–32.

50. Brindle RJ, Nunn PP, Githui W, Allen BW, Gathua S, Waiyaki P. Quantitative bacillary response to treatment in HIV-associated pulmonary tuberculosis. Am Rev Respir Dis. 1993;147(4):958–61. doi:10.1164/ajrccm/147.4.958.

51. Grzybowski S, Barnett GD, Styblo K. Contacts of cases of active pulmonary tuberculosis. Bull Int Union Tuberc. 1975;50(1):90–106.

52. Telzak EE, Fazal BA, Pollard CL, Turett GS, Justman JE, Blum S. Factors influencing time to sputum conversion among patients with smear-positive pulmonary tuberculosis. Clin Infect Dis. 1997;25(3):666–70.

53. Forte M, Maartens G, Rahelu M, Pasi J, Ellis C, Gaston H, et al. Cytolytic T-cell activity against mycobacterial antigens in HIV. AIDS. 1992;6(4):407–411. doi:10.1097/00002030-199204000-00008.

54. Kwan CK, Ernst JD. HIV and tuberculosis: A deadly human syndemic. Clin Microbiol Rev. 2011;24(2):351–376. doi:10.1128/CMR.00042-10.

55. Shen JY, Barnes PF, Rea TH, Meyer PR. Immunohistology of tuberculous adenitis in symptomatic HIV infection. Clin Exp Immunol. 1988;72(2):186–9.
