# Supplementary figures and images for: Estimating Mycobacterium tuberculosis transmission in a South African clinic: Spatiotemporal model based on person movements
Source: PLoS Comput Biol. 2025 Feb 18;21(2):e1012823. doi: 10.1371/journal.pcbi.1012823 (PMC11856658; doi:10.1371/journal.pcbi.1012823)

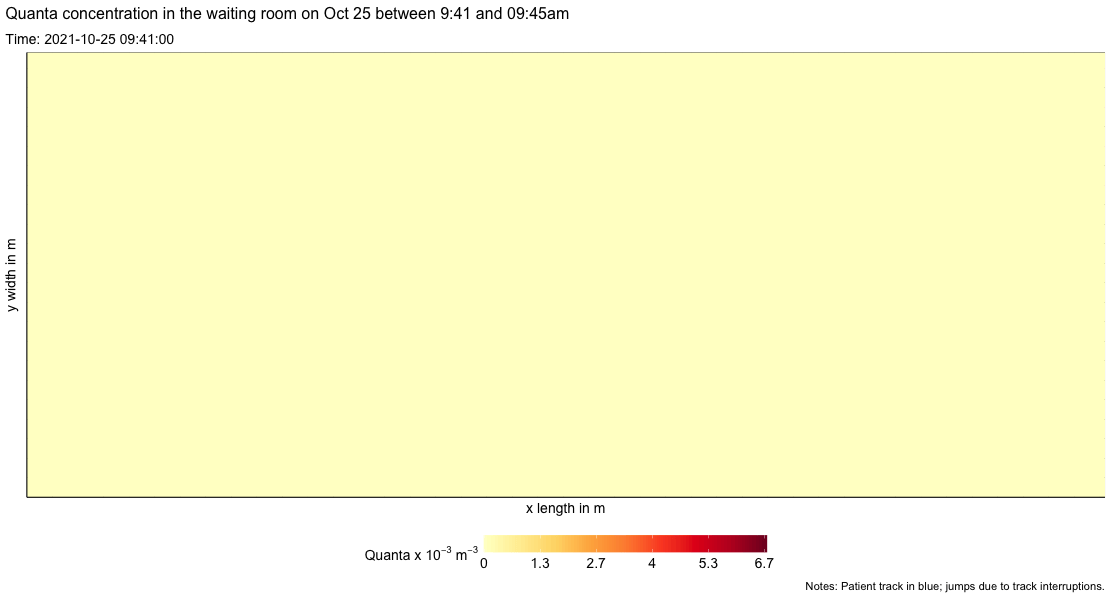

Supplement: S1 Video — (GIF) [file pcbi.1012823.s002.gif]
