# Supplementary material for: Estimating Mycobacterium tuberculosis transmission in a South African clinic: Spatiotemporal model based on person movements
Source: PLoS Comput Biol. 2025 Feb 18;21(2):e1012823. doi: 10.1371/journal.pcbi.1012823 (PMC11856658; doi:10.1371/journal.pcbi.1012823)
Supplement: Attachment — Submitted filename: STM - Response Letter.docx [file pcbi.1012823.s003.docx]

Point-by-point response letter

**Manuscript ID:** PCOMPBIOL-D-24-01011 **:**

**Manuscript title:** "Estimating Mycobacterium tuberculosis transmission in a South African clinic: Spatiotemporal model based on person movements"

Dear Editor, dear Reviewers,

Thank you for your constructive reviews of our manuscript. We appreciate the overall positive and helpful feedback and welcome the opportunity to submit a thoroughly revised version of our manuscript.

Below we provide a point-by-point response to each of your comments. Changes that we have incorporated during the revision are highlighted **in magenta** in the revised manuscript.

Yours sincerely,

Nicolas Banholzer and Lukas Fenner on behalf of all authors

# Response to Reviewer #1

**Comment 1.0:** In this manuscript, Banholzer et al. develop methods to quantify the spatiotemporal changes of risk within a clinic accounting for movement of individuals in the clinic, uncertainty in the number of infectious individuals and changes of environmental exposure as a function of heterogeneous shedding, exchange of air through ventilation and the corresponding diffusion of pathogens in the environment. This manuscript is very well written, and the methods described have potential to be used to study a wide variety of pathogens in many settings.

**Response 1.0:** Thank you very much for your positive assessment and helpful comments regarding our manuscript. All of the Reviewers’ and the Editor’s comments have been addressed below.

**Comment 1.1:** Comments:

1. Consider including a reference to Figure F from the appendix at the sentence “Four devices monitored indoor CO2 levels (CO2 Logger CL11, Rotronic, Bassersdorf, Switzerland) in the waiting room, corridor, and TB room (Figure 1), in parts per million (ppm) at 1-min intervals” in the Data collection part of the methods. There are a number of additional connections that can be made between the main text and the appendix.

**Response 1.1:** We have added a reference to Figure F (now Figure A) in S1 Appendix. Following your comments and those from Reviewer #2, we have expanded the number of links between the main text and the appendix and moved some details from the appendix to the main text:

- Discretization of the clinic space into a grid of cells (Methods, Spatiotemporal modeling approach, p. 9).
- Ventilation conditions in the clinic (Methods, Study design and setting, p. 3).
- Intraday CO_2_ levels and room occupancy (Methods, Data collection, p. 4; Results, p. 12).
- Room separation to facilitate modeling (Discussion, p. 18).

**Comment 1.2:** 2. There is some justification for simulating quanta separately for waiting room, corridor and TB room. Can you describe in more detail how the different areas (waiting room, corridor and TB room) were ventilated? How was air flow connected between the three areas? And what could have been the implications of treating them separately even though there may be some dependence between them?

**Response 1.2:** Rooms were ventilated by opening all doors and windows to increase natural ventilation. We have clarified the ventilation conditions in the manuscript (Methods, Study design and setting, p. 3):

*“The clinic was naturally ventilated by opening all doors and windows throughout the day.”*

As was described in the Appendix, the door between the waiting room and corridor was usually not closed, but room occupancy and CO_2_ levels varied between these areas. We considered the two areas to be separate to facilitate modelling, but recognized that there may be some flow of quanta between the waiting room and the corridor. We have noted and discussed the limitations of this assumption in the revised manuscript (Discussion, p. 18):

“*[...] the waiting room and corridor were treated as separate rooms to facilitate modeling and because we observed differences in room occupancy and time-varying CO_2_ levels, but the door between these areas was typically open, allowing aerosolized Mtb (quanta) to spread from one area to the other.*”

**Comment 1.3:** 3. In the description of the spatiotemporal risk model, it sounds like the risk is calculated by ‘cell’ by second throughout the day, but then this sentence (Methods; Statistical analysis) makes it sound like the risk is averaged over half days. “The average quanta concentration was computed by daytime (morning: 8 am to 12 am, afternoon: 12 am to 4 pm) and the risk of infection was summarized with the mean and 80%-credible interval (CrI)” Can you clarify what the temporal resolution of the risk that you used was to generate your results?

**Response 1.3:** We apologize for the confusion. We simulated the quanta concentration over time (in seconds) and space (per room and grid cell). In each simulation, we computed the individual risk of infection based on the cumulative spatiotemporal quanta exposure. We then computed the mean risk of infection per individual across simulations. The risk distribution was summarized across individuals with the mean and 80%-CrI. The risk distributions were compared between scenarios. As an additional result, we provided the average quanta concentrations by daytime across simulations to show the average quanta exposure. We have clarified our analysis in the revised manuscript (Methods, Statistical analysis, p. 10-11):

*“We modeled the spatiotemporal quanta concentration with 5,000 Monte Carlo simulations, considering uncertainty in modeling parameters (Section D in S1 Appendix). In each simulation, we computed the individual risk of infection based on the cumulative quanta exposure during the clinic visit. We computed the average quanta concentration by daytime (morning: 8am to 12am, afternoon: 12am to 4pm) and the mean risk of infection per clinic attendee across simulations. The scenario-specific infection risk distribution was shown with a histogram and summarized with the mean and 80%-credible interval (CrI).”*

**Comment 1.4:** 4. Figure F in Appendix Section D presents figures that illustrate CO2 levels by clinic area over time for each study day. There is very little description of this data, yet differences in the measurements during and between days seems worth describing, including discussing possible reasons for the observed temporal changes over time and by study day.

**Response 1.4:** CO_2_ levels vary with room occupancy and ventilations. Intra-day variations in CO_2_ levels primarily follow changes in occupancy. The clinic was most frequently visited during morning hours, hence the typical peaks before noon. Ventilation conditions may have varied between clinic days, explaining daily variations. We have explained this result in the revised manuscript (Results, p. 12).

“*CO_2_ levels varied slightly within and between clinic days, largely corresponding to changes in room occupancy during the day (Fig. A-B in S1 Appendix), and possibly small variations in ventilation conditions between days*”

Note that CO_2_ levels were low throughout the study, suggesting that the clinic was generally well ventilated.

**Comment 1.5:** 5. Appendix Section E.2 Pathogen specific factors – Are there other surrogate pathogens for which survival by temperature and humidity are known that could inform the assumptions of the model with Mtb?

**Response 1.5:** As outlined in the Appendix, the survival and dispersion of aerosols is further determined by physicochemical properties of the pathogen-carrying particles. Relative humidity is likely to affect the transport and viability of viruses in aerosols by modulating the rate of evaporation and the aerosol’s size. Temperature is likely to affect the stability of the proteins, lipids and genetic material of the pathogens. These relationships are complex and often pathogen-specific. To our knowledge (Appendix E.2), the impact of temperature and humidity on *Mtb* survival in bioaerosols is unclear and we are not aware of a perfect surrogate.

# Response to Reviewer #2

**Comment 2.0:** This paper presents a spatiotemporal model of airborne disease transmission, which extends the Wells-Riley equations by incorporating environmental, clinical and person movement data. The model is applied to simulate the infection risk of Mycobacterium tuberculosis in a South African clinic, where detailed environmental and movement data were gathered over five days during the COVID-19 pandemic. The authors use a Monte-Carlo simulation to consider the uncertainty in model parameters.

This paper is clearly written with nice graphics. The novelty of this work is highlighted by the combination of spatiotemporal models with real-world data, alongside the incorporation of uncertainty using a Monte Carlo approach. In addition, the authors have developed an open-source app to reconstruct the tracks of person movement, which will facilitate future studies in this field. I found this work a valuable contribution to the disease modelling and policymaking communities, and the methodology presented has the potential to be applied to other airborne diseases and a variety of indoor settings.

However, there are several issues in the mathematical model that require the authors’ attention. Addressing these issues may require updates to the simulation results and a revised discussion.

**Response 2.0:** Thank you very much for finding merit in our work. We appreciate your very detailed comments on our manuscript. We have accordingly revised our manuscript to address the issues you have raised (see below). We think that your feedback has greatly improved our manuscript.

**Comment 2.1:** Major comments:

1. Methods – quanta removal (units): It is weird that the inactivation rate of Mtb has a unit of quanta/h. It should have the unit of 1/h or 1/s, because the inactivation quanta number is also proportional to the local quanta concentration. The same issue applies to the air exchange rate (AER_t in Eq.2). For the second part of Eq. 2, we need to get a number before taking the exponential (and thus to obtain a fraction of the previous quanta concentration due to decay factors). With the current unit, we will get exp(quanta), which doesn’t make sense.

Although the unit used for the inactivation rate of Mtb is wrong, the values seem appropriate for the unit of 1/h. I checked one reference (Gannon et al., 2007, Res J Vet Sci), and I got an inactivation rate of 0.46/h instead of 0.46 quanta/h, presented in Fig. D in S1 Appendix. In this case, it is likely (and hopefully) that this unit issue won’t affect the results and the conclusion of this paper. The authors should verify their derivation of the inactivation rate and the air exchange rate, and correct the units in both the manuscript and appendix where necessary.

**Response 2.1:** Well spotted, thank you. The units for the quanta removal rates (viral inactivation and air exchange rate) were incorrectly reported as quanta/h rather than per hour. We have corrected the units for the quanta removal rates in the revised manuscript (Methods, Spatiotemporal modelling approach, p. 8-9) and appendix. We confirm that the model had already been correctly coded in the submitted version.

**Comment 2.2:** 2. Methods – quanta removal (gravitational settling): The authors set the gravitational settling rate to 0, citing the particles with Mtb have a settling velocity of 3.5e-5 to 1.5e-3 m/s. This sentence doesn’t make sense. For instance, if we take the maximum value 1.5e-3 m/s, the particles generated by an infectious person standing at 1.7m only take ~20 minutes to fall on the ground, which seems to dominate over other factors. Even if we assume that the settling velocity is at the order of 1e-4 m/s, it still yields ~0.2/h, which is comparable to the inactivation rate mentioned above. I would suggest the authors either include a gravitational settling rate, or acknowledge this limitation and provide a short discussion on how gravitational settling would affect the results.

**Response 2.2:** Thank you very much for pointing this out. We have addressed your comment by including the gravitational settling rate in the revised model. We approximated the settling velocity with a Gamma distribution with a mean of 0.0007675 m/s (the midpoint of the range from 3.5e^-5^ to 1.5e^-3^ m/s) and a standard deviation of 0.00037 m/s (range divided by 3.92). The Gamma distribution ensures non-negative settling velocities and places slightly more probability mass on slower settling velocities, consistent with the finding by Fennelly et al. (2020, doi:10.1016/S2213-2600(20)30323-4) that *Mtb* is more frequent in particles at the lower end of the size range from 1 to 7μm. In addition, we assumed a fixed drop height of 1.7m, resulting in a median gravitational settling rate of 1.5 h^-1^ (95%-CrI 0.5 h^-1^ – 3.5 h^-1^). We have revised our manuscript as follows (Methods, Spatiotemporal modelling approach, p. 8-9):

*“Particles with Mtb in the size range of 1 − 7μm^4^ have a settling velocity of 3.5 · 10^−5^ − 1.5 · 10^−3^ m s^−1^ in still air^22^, which we modeled using a Gamma distribution with mean 7.7 · 10^−4^ m s^−1^ and standard deviation 3.7 · 10^−4^ m s^−1^. Considering a drop height of 1.7 m, the median gravitational settling rate was assumed to be 1.5 h^−1^ (95%-CrI 0.5 h^−1^ – 3.5 h^−1^).”*

We updated our simulation results accordingly. The estimated risks of infection were generally reduced after taking the gravitational settling rate into account. Importantly, this change did not affect the conclusions we drew from our IPC scenario comparisons.

**Comment 2.3:** 3. Methods – Risk of infection (Eq. 2): Below Eq. 2, the authors define I_t as the number of infectious individuals in space. It seems the space here indicates a cell space c, instead of the space of the entire domain. In this case, I would suggest the authors to first introduce they discretised the space into cells, and then state explicitly that the Eq. 2 is for each cell ‘c’. In addition, the authors need to add subscript ‘c’ for N_t, N_(t-1), I_t and AER_t.

Furthermore, the units in the Laplacian don’t match, N_(t-1) is a concentration with quanta/m^3 while the second term gives quanta. I believe the q in the second term should be divided by the volume of a cubic cell. However, this depends on the mixing assumption made by the authors (see comment 5). The authors should check their model and code, and revise Eq. 2 accordingly.

**Response 2.3:** We apologise for the incorrect model description. In the revised manuscript, we have noted that we assume a vertically well-mixed airspace and that we have rasterized each 2-dimensional area into a grid of cells. Accordingly, we have revised Equation 2 and the surrounding paragraph (Methods, Spatiotemporal modelling approach, p. 9):

*“The quanta concentration was computed separately for the waiting room, corridor, and TB room. Each room was discretized into a grid of cubic cells, with each cell covering a squared area of 0.0625 m^2^. We assumed that the quanta concentration was vertically well-mixed and computed the quanta concentration N_r,c,t_ in room r and cell c at time t as*

 *where D_t_ is the diffusion constant (m^2^ s^−1^) varying by daytime, ∆ is the Laplace operator (second-order differential operator), I_r,c,t_ is the number of infectious individuals, V_r,c_ is the room-specific cell volume (m^3^), q is the quantum generation rate (quanta s^−1^), AER_r,t_ is the room-specific air change rate varying by daytime (s^−1^), λ is the bacterial inactivation rate (s^−1^), and k is the gravitational settling rate (s^−1^). We modeled the spatiotemporal quanta concentration during clinic hours from 8 am to 4 pm and updated it every second, corresponding to the frequency of the person-tracking data.”*

We note that the model had already been correctly coded in the submitted version.

**Comment 2.4:** 4. Methods – Risk of infection (mask efficiency): The authors didn’t consider the effects of masks on the inhalation of infectious aerosols in Eq. 3. While the authors have assumed a reduction in quanta generation by 75%, a similar reduction in inhalation should be applied. In the Discussion, the authors suggest that the masks are less effective in their case study, due to high ventilation rates. However, I believe this is because the authors didn’t consider the impact of masks on inhalation.

**Response 2.4:** There is broad consensus that tuberculosis is a strictly airborne disease. Therefore, public health authorities, including CDC and WHO, recommend FFP2 respirators for health care workers to prevent transmission and surgical face masks for patients with TB as source control to reduce the spread of infectious aerosols. Although surgical and community masks may also impact inhalation, this effect is likely to be negligible. Similarly, a mathematical modelling study of the effect of infection and control measures on *Mtb* transmission in healthcare settings (McCreesh et al., BMJ Global Health 2021, doi:10.1136/bmjgh-2021-007124) made the same assumption, and we compared our results with theirs (see Discussion, p. 17). In response to your comment, we have made our assumption about the impact of mask use explicit (Methods, Spatiotemporal modelling approach, p. 9–10):

*“In line with a previous modeling study^19^, we assumed that the mandatory use of community and surgical masks during our study had only effects on the exhalation of Mtb particles and no effect on Mtb infection of the person wearing the mask.”*

**Comment 2.5:** 5. Methods – Monte Carlo simulation (2D or 3D): In the appendix, the authors state that the aerosols diffuse in (x,y) direction. It is unclear how the authors calculate the quanta concentration along the room height. Did you assume the quanta are well-mixed vertically? The authors should clarify this in the manuscript. In addition, maybe use ‘…cubic cells, with each cell covering an area…’, since a cubic cell has a volume instead of an area.

**Response 2.5:** Thank you very much for the comment. Indeed, we assumed that the quanta are well-mixed vertically. We have specified this assumption in the revised manuscript and adopted your description of cubic cells (Methods, Spatiotemporal modelling approach, p. 9).

**Comment 2.6:** Minor comments:

6. Introduction – 2nd paragraph: A minor suggestion: rephrase ‘because infectious individuals are more likely to visit clinics’ to ‘where infectious individuals are more likely to visit’.

**Response 2.6:** Thank you, we have revised this sentence accordingly.

**Comment 2.7:** 7. Methods – quanta generation – 1st paragraph: The authors allowed a maximum delay of 15 minutes between an individual was detected in the registration area and their entry in the database. This seems quite long for a small clinic. I was wondering why the authors set the threshold as 15 minutes?

**Response 2.7:** We had discussed the routine patient flow (including the registration process) and the threshold for matching with the local study team and the clinic staff, and agreed on the 15-minute threshold. Empirically, we matched 20 clinical records of TB patients (diagnosed and suspected TB cases) with patient tracks. The median delay between the track and the entry into the database was 2 minutes and the maximum was just under 10 minutes.

**Comment 2.8:** 8. Results – 2nd paragraph & S2 video: It would be helpful to add the dimensions along the two axes, and represent the location of the infectious individual using a distinct colour in the video.

**Response 2.8:** Great idea, we have added the dimensions and the location and path of the individual is now represented with a blue-colored line.

**Comment 2.9:** 9. Results – Fig. 4: In Fig. 4b, the vertical axis should be number of people instead of number of tracks? In Fig. 4c, the outliers for TB room seem a bit too many? Some explanation for this would be helpful.

**Response 2.9:** With regards to Fig. 4b: Occasionally the video sensors may have been unable to detect a person or mistook a person for an object, hence our use of the descriptive label “number of tracks”. We have omitted this detail and use ‘number of people’ as the new y-axis label. With regards to Fig. 4c: The TB room is rarely occupied because it is reserved for diagnosed or suspected TB patients. Thus, the time spent in the TB room is zero for most patients, but sometimes much higher (as indicated by the many outliers in the box plot). Following your suggestion, we have added a brief explanation to the revised manuscript (Results, p. 12):

*“Time spent in the corridor and TB room varied considerably as many patients just walked through the corridor and the TB room was reserved primarily for diagnosed or suspected TB patients.”*

**Comment 2.10:** 10. Appendix: I appreciate the authors’ efforts in preparing a detailed appendix, which includes plenty of useful information. However, for a mathematical biology journal like PLOS Computational Biology, it might be useful to include key modelling assumptions and numerical details in the main text (instead of the appendix), which would improve readability and accessibility.

**Response 2.10:** Thank you, we appreciate the opportunity to include more information from the appendix in the revised manuscript. Following your comments and those from Reviewer #1, we have moved several details from the appendix to the main text:

- Discretization of the clinic space into a grid of cells (Methods, Spatiotemporal modeling approach, p. 9).
- Ventilation conditions in the clinic (Methods, Study design and setting, p. 3).
- Intraday CO_2_ levels and room occupancy (Methods, Data collection, p. 4; Results, p. 12).
- Room separation to facilitate modeling (Discussion, p. 18).
